# Economic burden of recurrent vulvovaginal candidiasis in Uganda: a cost-of-illness analysis

**DOI:** 10.1016/j.ijregi.2025.100680

**Published:** 2025-06-05

**Authors:** Bwambale Jonani, Felix Bongomin

**Affiliations:** 1 Department of Immunology and Molecular Biology, School of Biomedical Sciences, College of Health Sciences, Makerere University, Kampala, Uganda; 2 Department of Clinical Laboratories, Sebbi Hospital, Wakiso district, Uganda; 3 Department of Medical Microbiology and Immunology, Faculty of Medicine, Gulu University, Gulu, Uganda

**Keywords:** Recurrent vulvovaginal candidiasis, Economic burden, Uganda

## Abstract

**Objectives:**

Recurrent vulvovaginal candidiasis (rVVC) affects 6-9% of women worldwide, yet its economic impact in resource-limited settings remains poorly understood. This study estimated the economic burden of rVVC in Uganda from a societal perspective.

**Methods:**

We used a cost-of-illness framework using secondary data sources. We modeled the rVVC prevalence by adjusting for three major epidemiologic risk factors: HIV infection, hormonal contraceptive use, and broad-spectrum antibiotic exposure. Direct medical costs were calculated using Uganda’s health care system pricing, whereas indirect costs were estimated using local wage data. Costs were adjusted for pharmaceutical markups (50-60%) and health-specific inflation (6.0%). Prevalence was determined using epidemiologic data adapted to Uganda’s female population aged 15-49 years. Sensitivity analyses addressed uncertainty in prevalence rates, health care–seeking behaviors, episode frequency, and wage variability.

**Results:**

The annual economic burden of rVVC in Uganda was estimated at Uganda shillings 280.077 billion (US $75.696 million), equivalent to approximately 11.22% of the total health care expenditure. Direct medical costs accounted for 71.5% of the total. The average annual cost per patient was Uganda shillings 260,082 (US $70.29), which is approximately 7.0% of per-capita gross domestic product. Health care–seeking behavior and prevalence were the most influential parameters in sensitivity analyses.

**Conclusions:**

rVVC imposes a substantial economic burden on Ugandan women and the health care system. Our findings highlight the need for enhanced screening, improved diagnostics and therapeutic approaches, and preventive strategies.

## Highlights

• Recurrent vulvovaginal candidiasis (rVVC) costs Uganda US $75.696 annually, which is 11.22% of health spending.
• Direct medical costs account for 71.5% of the total rVVC burden.
• The annual cost per rVVC patient is US $70.29 or 7% of per-capita gross domestic product.
• Prevalence and care-seeking drive cost; prevention is essential.

## Introduction

Vulvovaginal candidiasis (VVC) is a common genital yeast infection that affects up to 75% of women at least once during their lifetime [1,2]. Although most cases are acute and respond well to topical or oral antifungal treatment, approximately 6-9% of women worldwide develop recurrent VVC (rVVC), defined as four or more symptomatic episodes within a 12-month period [3]. Despite its high prevalence and significant impact on quality of life, the economic burden of rVVC remains poorly quantified, particularly, in resource-limited settings. rVVC substantially affects quality of life of the affected women and psychological well-being. Beyond the physical symptoms of pruritus, soreness, and discomfort during sexual intercourse, women with rVVC often report loss of confidence and self-esteem, inability to perform normal physical activities, and difficulties with their sexual life and intimate relationships [[4], [5], [6]]. These impacts translate into economic consequences through health care utilization, medication costs, and productivity losses.

The global antifungal market for gynecologic products is estimated at $600 million, with a substantial proportion of fluconazole ($242 million in 2013) and itraconazole ($350 million in 2011) sales attributed to VVC [7,8]. However, there are limited data separating the economic impact of acute VVC vs rVVC, particularly, in low-resource settings. In Uganda, the burden of rVVC remains largely unexplored. The health care system faces multiple challenges, including limited diagnostic capacity, variable access to antifungal medications, and out-of-pocket financing that creates financial barriers to care. With approximately 34.09% of health care expenditure coming from out-of-pocket payments [9], conditions such as rVVC may impose significant financial strain on affected women and their households. In this study, we estimated the economic burden of rVVC in Uganda using a cost-of-illness approach with secondary data sources.

## Methods

### Study design

We used a cost-of-illness framework using secondary data sources to quantify the economic burden of rVVC in Uganda. We adopted a societal perspective, incorporating direct and indirect costs to provide a comprehensive assessment of disease impact. The estimation of the number of rVVC cases in Uganda followed the Leading International Fungal Education (LIFE) program method. The LIFE method is a deterministic modeling approach that estimates the burden of serious fungal diseases in a specific country by applying disease-specific prevalence or incidence rates, sourced from global, regional, or local literature to national population and risk group data [10].

### Modeling prevalence of recurrent vulvovaginal candidiasis and assumptions

To estimate the national prevalence of rVVC among Ugandan women aged 15-49 years, we applied population-weighted adjustments to the global base prevalence of 6.0% [2], and the LIFE burden model [10]. Adjustments were made for three major epidemiologic risk factors that elevate rVVC likelihood in Uganda: HIV infection, hormonal contraceptive use, and broad-spectrum antibiotic exposure. HIV prevalence among women of reproductive age in Uganda is estimated at 6.6% [11]. The odds of symptomatic VVC are four- to eight-fold higher in women infected with HIV, particularly, those with high plasma viral load or clusters of differentiations counts <200 cells/mm^3^ [12]. To avoid overestimation, we conservatively modeled a 2.5-fold increase over the baseline, assuming 15% rVVC prevalence in this subgroup. Modern contraceptive use in Uganda is approximately 53.2% among women aged 15-49 years, with hormonal methods being the most used [13]. Oral contraceptives are estimated to increase the odds of VVC by 2.3 and injectable or implants by 8.7 [14]. Based on this, we applied a conservative adjusted prevalence of 8.5% for contraceptive users aged 15-49 years. Antibiotic exposure is also widespread in Uganda, with 41% of the prescribed antibiotics dispensed over the counter [15]. Antibiotic use alters the vaginal microbiota, increasing susceptibility to *Candida* overgrowth, with a 3.33-fold higher risk of *Candida* colonization post-antibiotic therapy [16]. Moreover, 34% of women using antibiotics develop recurrent episodes of VVC [17]. We therefore modeled a 9% rVVC prevalence among antibiotic users a conservative estimate below the likely clinical burden, accounting for variability in drug type, frequency, and host susceptibility (Table 1). Population-level adjustments were calculated using the following formula:

$$\begin{array}{ccc} Adjusted\;prevalence & = & Base\;prevalence+\sum Subgroup\;proportion\;x\\ & & \left(subgroup\;prevalence-base\;prevalence\right) \end{array}$$

**Table 1:** Population-weighted adjustment to rVVC prevalence estimate in Uganda.

Risk factor	Subgroup size (% of population)	Subgroup rVVC prevalence (%)	Effect size/justification	Incremental a to national prevalence (%)
Base global prevalence	100.00	6	Global estimate by Leading International Fungal Education/Global Action for Fungal Infections (GAFFI) and Denning *et al.*	6
HIV+ women	6.60	15	OR 4-8 for symptomatic VVC; modelled as 2.5 × baseline	0.594
Hormonal contraceptive users	53.20	8.5	OR 2.3-8.7; modelled conservatively	1.33
Antibiotic users	41.00	9	Risk ratio = 3.33 post-antibiotics; 34% recurrence with antibiotics as trigger	1.23
Total adjusted prevalence				≈ 9.154

OR, odds ratio; rVVC, recurrent vulvovaginal candidiasis.

### Study population and prevalence estimation

Previous estimate of the prevalence of rVVC by Bongomin *et al*. [18] applied the LIFE method on the 2020 estimated population of Uganda (45,741,000) and the global burden of rVVC by 2018 (6%) [2] to estimate 656,340 cases of rVVC in Uganda. We updated this estimate using the May 2025 The National Population and Housing Census 2024 final report figure [19], to estimate the current number of rVVC cases in Uganda, stratified according to age group. The target population comprised women of reproductive age (15-49 years) in Uganda, estimated at 11,764,068 (Supplementary Table 1).

### Treatment pathway mapping

**Figure 1:**
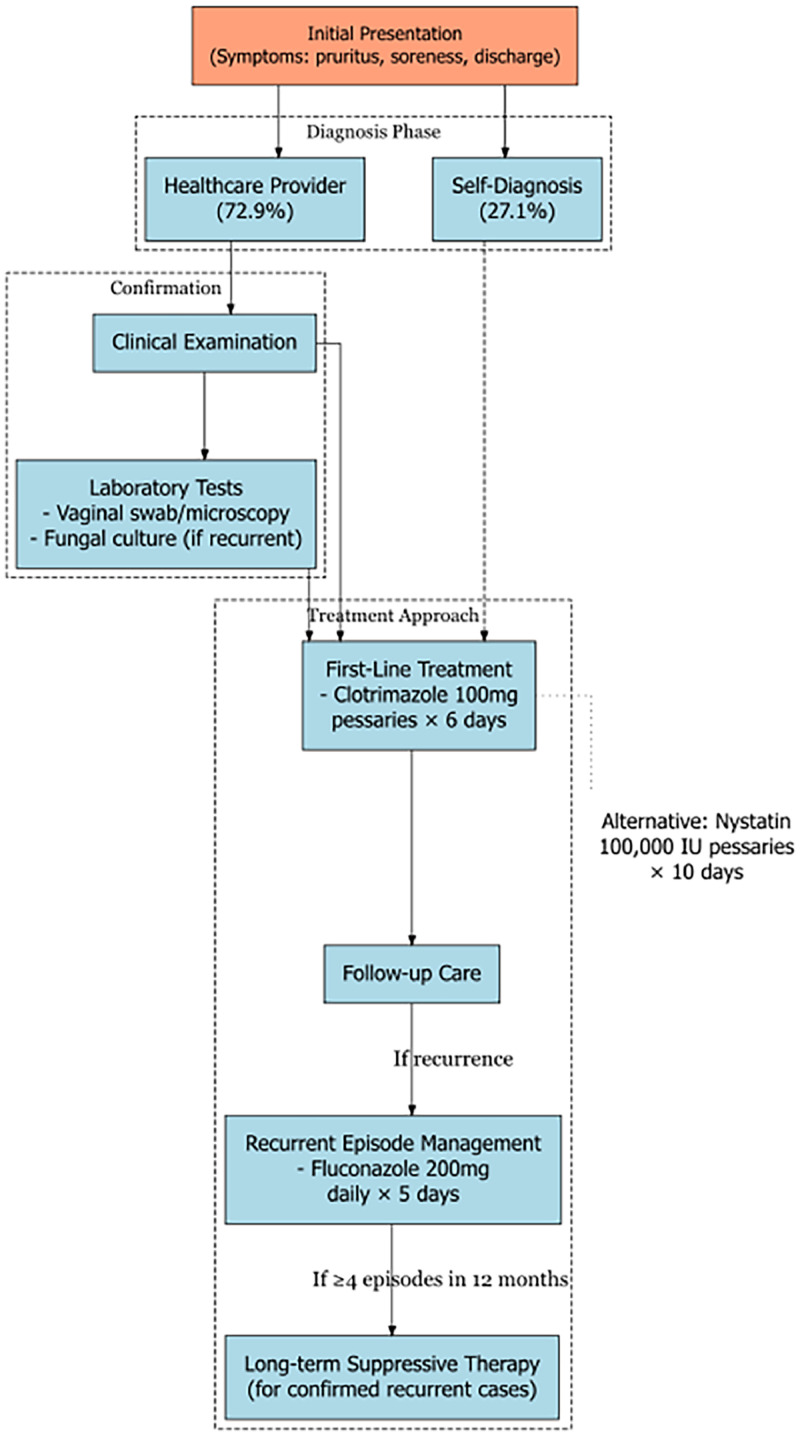
Clinical management pathway for recurrent vulvovaginal candidiasis in Uganda.

We defined the standard clinical management pathway for rVVC by synthesizing the Uganda Clinical Guidelines 2023 and the British Association for Sexual Health and HIV guideline where local guidelines were limited (Figure 1). This approach accounts for health care–seeking behaviors observed in similar settings, where approximately 72.9% of women obtain a diagnosis through a health care provider, whereas 27.1% self-diagnose and self-treat [17].

The pathway encompasses initial consultation and diagnosis, laboratory confirmation (where available), first-line treatment with antifungal agents, follow-up care and management of recurrent episodes, and long-term suppressive therapy for confirmed recurrent cases.

### Cost data collection and transfer methodology

Drug costs were obtained from the Joint Medical Stores of Uganda price list for fluconazole, nystatin, and clotrimazole (Supplementary Table 2). We also considered local health care system markup structures, applying a 50-60% markup for retail pharmaceuticals based on estimates from the Medicines Transparency Alliance [20].

### Cost components

We categorized costs into direct costs, direct non-medical costs, and indirect costs (Table 2). For items without direct local pricing, we applied conversion factors using multiple approaches. We used the gross domestic product (GDP) per-capita ratio between Uganda (US $1002) and reference countries, with data sourced from World Bank national accounts and Organization for Economic Co-operation and Development National Accounts data files [21]. We incorporated health care–specific purchasing power parity indexes obtained from the Organization for Economic Co-operation and Development (2023) [22].

**Table 2:** Resource use and annual cost of managing vaginal candidiasis in Uganda (adjusted for 6% inflation).

Category	Unit cost (UGX)	Units per course	Course cost (UGX)	Annual utilization	Annual cost (UGX)	Inflated annual cost (UGX)	Inflated annual cost (US$)	Narration
**Direct medical costs**								
Initial consultation	10,000	1	10,000	1	10000	10,600	2.86	One consult per episode
Vaginal swab/microscopy	15,000	1	15,000	2	30000	31,800	8.59	To detect fungal elements
Fungal culture (recurrent)	50,000	1	50,000	2	100000	106,000	28.65	Used in two of four episodes
**First-line treatment**								
Clotrimazole pessaries	303	6	1820	1	1820	1929	0.52	First-line, acute cases
**Recurrent treatment**								
Fluconazole tablets	236	5	1180	3	3540	3752	1.01	Recurrent episodes two to four
Follow-up consultation	10,000	1	10,000	3	30000	31,800	8.59	For episodes two to four
**Direct non-medical costs**								
Transportation (round-trip)	10,000	1	10,000	4	40000	42,400	11.46	Travel to facility

UGX, Uganda shillings.

### Economic burden

Patient-level costs were calculated by multiplying the frequency of health care resource utilization by the corresponding unit costs. The standard treatment pathway for acute and recurrent VVC was valued based on typical utilization patterns. All costs were calculated in Ugandan shillings (UGX) and converted to US $ using the average 2025 exchange rate (1 US $ is approximately 3700 UGX). We applied a 6.0% health-specific inflation rate based on the consumer price index for health services in Uganda [23] to ensure contemporary relevance of historical cost data.

## Productivity loses

For productivity losses, we adapted the estimates from the study on the subjective health status and health-related quality of life among women with rVVC [4], which estimated that approximately 33 hours are lost per year due to rVVC symptoms. This was valued using gender and education-specific wage data for Uganda [24].

### Population-level extrapolation

We extrapolated patient-level costs to the national population using our prevalence estimates applied to the female population aged 15-49 years.

### Sensitivity analyses

Worldwide and in Uganda, there is a lot of uncertainty surrounding key parameters such as rVVC prevalence, health care–seeking behavior, episode frequency, and wage variability. To address this, we conducted sensitivity analyses by applying a ±50% variation to each parameter. This approach was based on the observed global variability in rVVC prevalence (6-9%), which represents a 50% increase from the base case of 6% [2]. Our goal was to capture a realistic range of outcomes for the population. The key model parameters and their ranges were as follows:

1. Prevalence of rVVC: We varied this from 4.6% to 13.7% (±50%) around the modeled base value of 9.154%.
2. Health care–seeking behavior: We ranged this from 36.5% to 109.4% (±50%) around a base value of 72.9%, reflecting the variability in treatment access and health-seeking behavior [17].
3. Number of rVVC episodes per year: We varied this from two to six episodes annually (±50%) around a base case of 4, consistent with the clinical definition [25].
4. Wage rates by education level and employment formality: We varied these by ±50% from base case values to reflect income disparities across socioeconomic groups, as described in [24].

## Results

### Prevalence and demographic distribution

**Figure 2:**
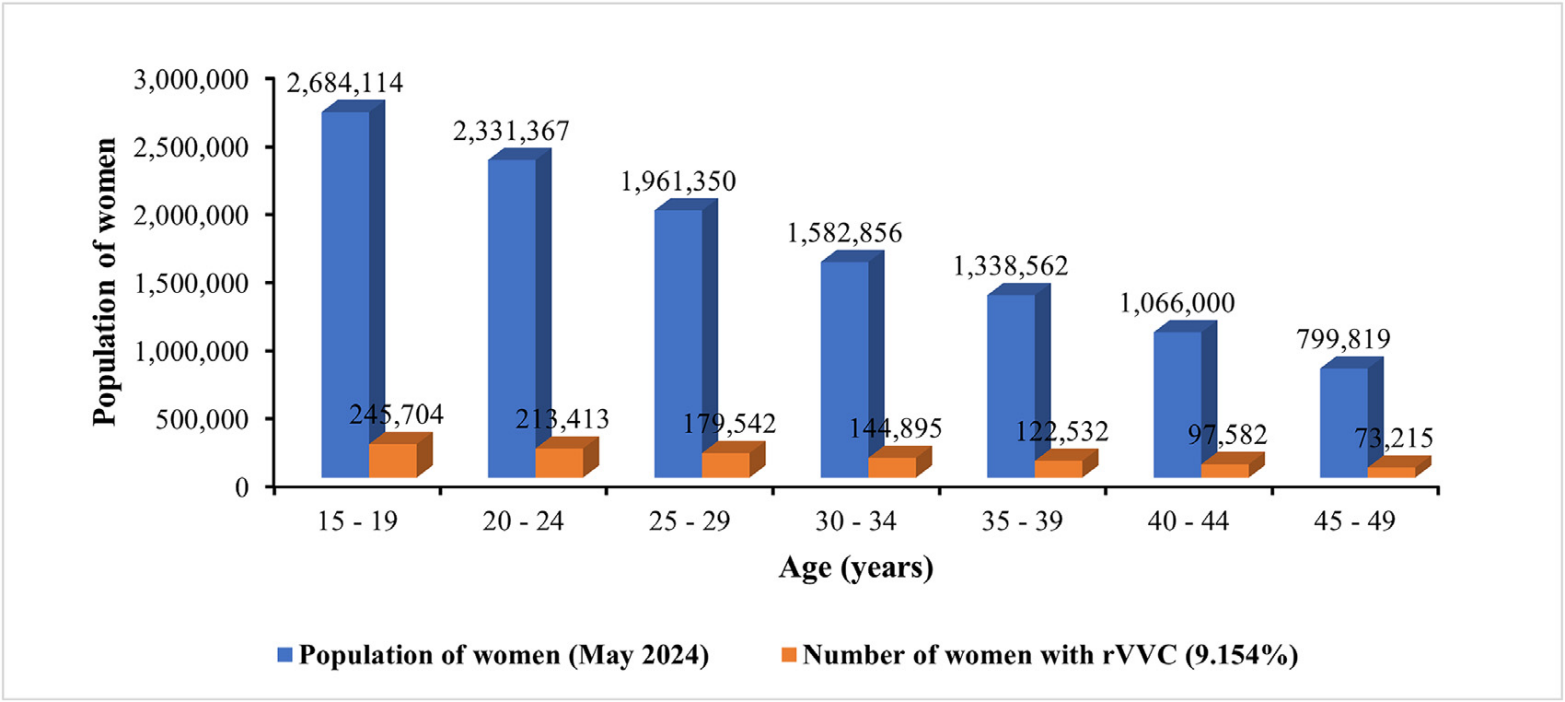
Estimated number of women with recurrent vulvovaginal candidiasis by age group in Uganda.

An estimated 1,076,833 women of reproductive age in Uganda experience rVVC annually. The age distribution follows the population structure, with the highest relative numbers in the younger age groups (Figure 2).

### Per-patient economic burden

The average annual economic burden per patient with rVVC in Uganda is 260,082 UGX ($70.29) (Table 3). Direct medical costs constitute 71.5% of total costs, followed by indirect costs at 16.3% and direct non-medical costs at 12.2%.

**Table 3:** Annual per-patient economic burden of rVVC in Uganda.

Cost category	Amount (UGX)	Amount (US$)	Percentage of total
**Direct medical costs**			
Consultations	42,400	11.46	16.3%
Diagnostic tests	137,800	37.24	53.0%
Medications	5682	1.54	2.2%
**Subtotal**	**185,882**	**50.24**	**71.5%**
**Direct non-medical costs**			
Transportation	42,400	11.46	16.3%
**Subtotal**	**42,400**	**11.46**	**16.3%**
**Indirect costs**			
Lost productivity at work	31,800	8.59	12.2%
**Subtotal**	**31,800**	**8.59**	**12.2%**
**Total annual cost per patient**	**260,082**	**70.29**	**100.0%**

rVVC, recurrent vulvovaginal candidiasis; UGX, Uganda shillings.

Exchange rate: US $1 = 3700 UGX.

The economic burden per patient represented approximately 7.01% of the annual per-capita GDP of Uganda ($1002 in 2023) [21], highlighting the substantial financial impact of rVVC on affected women.

### National economic burden

Extrapolating the per-patient costs to the estimated 1,076,833 women with rVVC in Uganda, the total national economic burden of rVVC was estimated to be UGX 280.077 (US $75.696 million). This total annual economic burden of rVVC in was approximately 11.22% of the total health expenditure budget of 2.496 trillion UGX [26], with indirect medical costs representing the largest proportion of expenditures related to its clinical management.

### Sensitivity analysis

**Figure 3:**
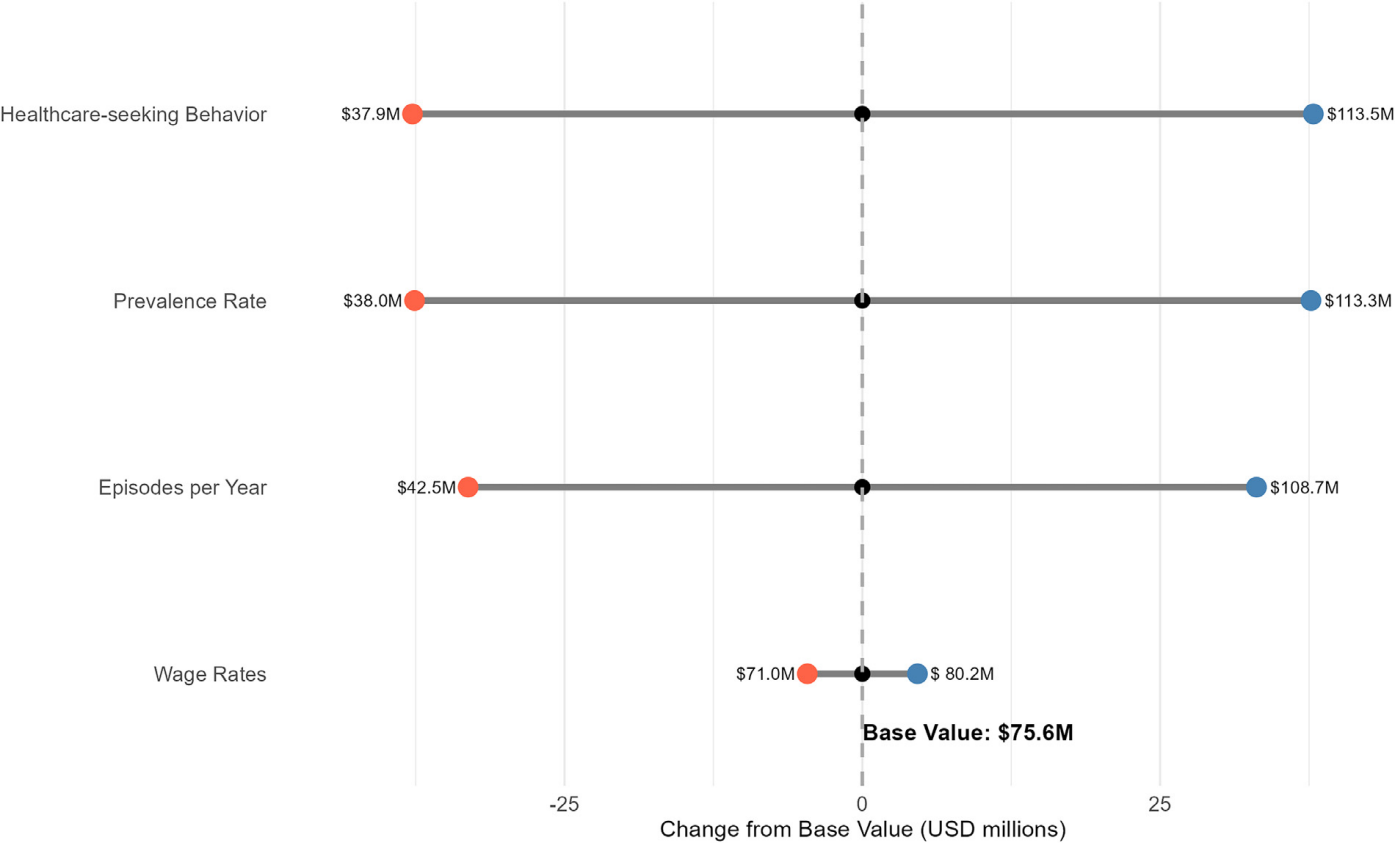
Sensitivity analysis of economic burden of rVVC in Uganda (±50% parameter variation).

Varying health care–seeking behavior and prevalence exerted the greatest influence on the national economic burden, with both having an impact range of ±$36.6 million (Figure 3).

rVVC, recurrent vulvovaginal candidiasis.

## Discussion

To the best of our knowledge, we provide the first estimate of the economic burden of rVVC in Uganda, revealing a substantial impact on individual women and the health care system. Our findings indicate that rVVC costs the Ugandan economy approximately UGX 280.077 billion (US $75.696 million) annually, with individual women bearing an average cost of UGX 260,082 (US $70.29) per year.

The economic burden of rVVC is disproportionately high relative to Uganda’s per-capita GDP, with annual per-patient costs representing approximately 7.0% of the average annual income. This highlights the significant financial strain placed on affected women, considering that 34.09% of health care expenditure in Uganda comes from out-of-pocket payments [27]. For many women, these costs may represent a substantial financial barrier to seeking appropriate care, potentially leading to self-treatment with suboptimal therapies or enduring symptoms without medical intervention.

Direct medical costs constituted the largest proportion (71.5%) of the total economic burden, reflecting the resource-intensive nature of diagnosing and treating recurrent episodes. Diagnostic tests alone accounted for 53.0% of direct medical costs, highlighting the importance of laboratory confirmation in managing rVVC. This cost distribution pattern may be influenced by several factors specific to the Ugandan health care context, including the structure of health care financing, limited laboratory capacity necessitating referrals for specialized testing, and the relative cost of medical services compared with local wage rates. Although our analysis captures the monetary value of productivity losses (12.2% of total costs), this proportion might be affected by the challenges in comprehensively measuring informal labor productivity and the socioeconomic status of affected women in Uganda.

The productivity impact may extend beyond the measurable hours of work missed, affecting women’s overall economic participation and advancement opportunities. In addition, our analysis was unable to fully capture the economic consequences of psychological distress and effects on personal relationships, which have been documented in qualitative studies of women with rVVC [[28], [29], [30], [31], [32]].

Our sensitivity analyses demonstrated that health care–seeking behaviors and prevalence estimates have the greatest influence on the national economic burden estimates. The wide variation in these parameters underscores the need for more robust epidemiologic data specific to the Ugandan context. The prevalence of rVVC may vary significantly across different demographic and socioeconomic groups, and health care–seeking patterns are influenced by multiple factors, including accessibility, affordability, and awareness of treatment options.

The economic burden of about US $75.6 million annually in Uganda demonstrates a significant impact on women’s health and the health care system. Emergency care for pregnancy-related complications, such as postpartum hemorrhage, a leading cause of maternal mortality, costs a median of US $17.25 per case at regional referral hospitals [33]. Considering the high incidence of these complications, total health care system costs likely reach tens of millions of dollars annually. Unsafe abortion and post-abortion care cause approximately US $13.9 million in annual health system costs, with this figure rising to US $20.8 million if the health system satisfies all care demand [34]. Obstetric fistula repair costs around US $378 per procedure and effectively reduces disability-adjusted life-years [35]. Together, these figures show that rVVC’s economic burden rivals or exceeds several well-known reproductive health challenges in Uganda, highlighting the need to prioritize rVVC in public health programs to reduce its financial and health impact on Ugandan women.

Our findings have important implications for health care policy and resource allocation. The Ministry of Health in Uganda should prioritize integrating screening and management of rVVC into existing antenatal health programs. This can be achieved by allocating dedicated funds to cover point-of-care *Candida* test kits and maintenance fluconazole therapy. To further improve the management of RVVC, policymakers should expand the Essential Medicines List to include alternative antifungal agents such as itraconazole. Importantly, a tiered care approach should be implemented, which includes strengthening diagnostic capacity at health center III and IV levels through training programs and provision of testing equipment. This should be accompanied by establishing referral pathways to regional hospitals for recurrent cases. Furthermore, the government should leverage Uganda’s successful community health care model and develop community-based education programs targeting village health teams. These programs should focus on improving early recognition and appropriate referral of RVVC cases.

There were several limitations to this analysis. We relied on secondary data sources and the LIFE program method rather than primary data collection, which may not fully capture the nuances of health care utilization patterns and costs specific to rVVC in Uganda. The prevalence estimate is based on international literature adjusted to the Ugandan context, which introduces uncertainty in the true disease burden. Our productivity cost estimates may not adequately capture the informal labor market, which constitutes a significant proportion of female employment in Uganda. Finally, our analysis does not account for all potential comorbidities or complications associated with rVVC, which may further contribute to the economic burden.

Future research should focus on collecting primary data on the prevalence and economic impact of rVVC in Uganda, including direct measurement of health care utilization patterns and out-of-pocket expenses. Studies exploring the intersection of rVVC with other reproductive health conditions and HIV would provide valuable insights into the comprehensive burden of fungal infections in women. In addition, economic evaluations of different management strategies, including prophylactic approaches, could inform more efficient resource allocation for this common but often overlooked condition.

## Conclusion

We demonstrate that rVVC imposes a substantial economic burden of UGX 280.077 billion on the Ugandan health care system and affected women. We provide evidence to support increased attention to fungal infections within women’s health programs and highlight the need for affordable and accessible diagnostic and treatment options. Addressing the burden of rVVC has the potential to improve health outcomes and economic well-being for thousands of Ugandan women.

## Declarations of competing interest

The authors have no competing interests to declare.

## Appendix Supplementary materials

Supplementary file Supplementary File 1. Includes Supplementary Table 1 (target population of women aged 15-49 years in Uganda) and Supplementary Table 2 (unit drug costs from Joint Medical Stores for fluconazole, nystatin, and clotrimazole).pdf. Image, application 1

## Footnotes

Supplementary material associated with this article can be found, in the online version, at doi:10.1016/j.ijregi.2025.100680.
