# Supplementary material for: Economic burden of recurrent vulvovaginal candidiasis in Uganda: a cost-of-illness analysis
Source: IJID Reg. 2025 Jun 5;16:100680. doi: 10.1016/j.ijregi.2025.100680 (PMC12221850; doi:10.1016/j.ijregi.2025.100680)
Supplement: Supplementary file 1 — Supplementary file Supplementary File 1. Includes Supplementary Table 1 (target population of women aged 15-49 years in Uganda) and Supplementary Table 2 (unit drug costs from Joint Medical Stores for fluconazole, nystatin, and clotrimazole).pdf. [file mmc1.pdf]

Supplementary table 1. Female Population in Uganda by Age Group (2024)

Age group (Years)	Population
15 - 19	2,684,114
20 - 24	2,331,367
25 - 29	1,961,350
30 - 34	1,582,856
35 - 39	1,338,562
40 - 44	1,066,000
45 - 49	799,819
Total (15 - 49 years)	11,764,068

Source: Uganda national population and housing census 2024 final report volume 1.

Supplementary table 2. Estimated costs associated with vaginal candidiasis in Uganda

Cost Category	Component	Unit Cost (UGX)	Unit Cost (USD)	WHO-CHOICE Estimate (I$)	Notes
**Direct Medical Costs**	Initial consultation	10,000	2.70	2.86 – 3.26	Average cost for primary healthcare visit; WHO-CHOICE for health centre / primary hospital outpatient visit
	Vaginal swab/microscopy	15,000	4.05	-	Demonstration of fungal elements by microscopy
	Fungal culture	50,000	13.51	-	Species identification and AFST for recurrent cases
	Medication costs	Varies	Varies	-	
	Over-the-counter treatments	5,000	1.35	-	Self-medication prior to seeking care
	Follow-up visits	10,000	2.70	2.86 – 3.26	No costs for revisit within 30 days; aligned with WHO-CHOICE estimates
**Direct Non-Medical Costs**	Transportation	10,000	2.70	-	Estimated average round-trip to a health facility
**Indirect Costs**	Absenteeism	-	-	-	6 hours per episode [2]
	Reduced productivity at work	-	-	-	Estimated at 33 hours annually [2]
	Value of lost work time	180,000/month	48.65/month	-	Based on wage data for Ugandan women [16]
	Hourly wage rate	909	0.25	-	Based on 198 working hours/month
	Cost of absenteeism per episode	5,455	1.47	-	6 hours × hourly wage rate
	Annual cost of reduced productivity	30,000	8.11	-	33 hours × hourly wage rate

Direct medical costs were calculated using current Ugandan healthcare prices in UGX and USD. WHO-CHOICE estimates were referenced to validate consultation costs and provide PPP-adjusted comparisons. WHO-CHOICE cost estimates represent modelled average costs per outpatient visit at different facility levels (e.g., health centres, primary/secondary hospitals), reported in 2010 International Dollars (I$), which adjust for purchasing power differences across countries.
